# Integrated Assessment of Sarcopenia in Patients with Gastric Cancer Using Deep Learning and Radiomics

**DOI:** 10.1002/jcsm.70377

**Published:** 2026-09-14

**Authors:** Huaiqing Zhi, Jingwei Zheng, Hao Chen, Xinxin Yang, Zhixuan Jiang, Ang Shi, Dongze Wu, Linxi Lv, Jinnuo He, Xiaodong Chen, Bujian Pan, Xian Shen, Weiteng Zhang

**Affiliations:** ^1^ Department of General Surgery The First Affiliated Hospital of Wenzhou Medical University Wenzhou Zhejiang Province China; ^2^ Department of Clinical Nutrition The First Affiliated Hospital of Wenzhou Medical University Wenzhou Zhejiang Province China; ^3^ Department of General Surgery The Second Affiliated Hospital and Yuying Children's Hospital of Wenzhou Medical University Wenzhou Zhejiang Province China; ^4^ Zhejiang Key Laboratory of Intelligent Cancer Biomarker Discovery and Translation The First Affiliated Hospital of Wenzhou Medical University Wenzhou Zhejiang Province China; ^5^ NHC Key Laboratory of Clinical Nutrition and Intervention (The First Affiliated Hospital of Wenzhou Medical University) Wenzhou Zhejiang Province China

**Keywords:** deep learning, gastric cancer, radiomics, sarcopenia

## Abstract

**Background:**

Sarcopenia is a common condition in patients with gastric cancer that is associated with poor survival and adverse clinical outcomes. Conventional diagnostic approaches are time‐consuming, limiting large‐scale clinical implementation. This study aimed to develop and validate a multimodal model that integrates clinical variables, radiomics features and deep learning features.

**Methods:**

A total of 1067 patients with gastric cancer from two medical centres were included. The diagnosis of sarcopenia required low grip strength and a low skeletal muscle index, whereas severe sarcopenia additionally required low gait speed. Radiomics features were extracted from computed tomography images at the third lumbar vertebral level to construct radiomics models. The 2D and 2.5D deep learning models were developed using the ResNet50 architecture. A transformer‐based multimodal model integrating clinical variables, radiomics features and 2.5D deep learning features was developed and termed the sarcopenia model, while an XGBoost fusion model integrating the same three modalities was constructed as a comparator. Model performance was evaluated in the training, validation and external test sets using receiver operating characteristic and decision curve analyses.

**Results:**

Among the 1067 patients, 782 (73.3%) were male and 285 (26.7%) were female, with no significant difference in gender distribution across the training, validation and external test sets (*p* = 0.34). Kaplan–Meier survival analysis demonstrated that the overall survival of patients with sarcopenia was significantly worse than that of patients without sarcopenia (*p* < 0.05). Among single‐modality models, the radiomics model achieved AUCs of 0.85 and 0.80 in the validation and external test sets, respectively. The 2.5D deep learning model outperformed the 2D model, yielding AUCs of 0.81 and 0.83 in the validation and external test sets. The XGBoost model achieved AUCs of 0.85 and 0.86 in the validation and external test sets, respectively. In the sarcopenia model, the AUCs for identifying sarcopenia were 0.95, 0.87 and 0.89 in the training, validation and external test sets, respectively, while those for identifying severe sarcopenia were 0.93, 0.85 and 0.84.

**Conclusions:**

The sarcopenia model integrating clinical variables, radiomics features and deep learning features enables the accurate identification of sarcopenia and severe sarcopenia in patients with gastric cancer and may support clinical screening and risk stratification.

## 1 Introduction

Gastric cancer (GC) remains a leading malignancy worldwide, imposing a substantial burden on public health [1]. Driven by systemic metabolic alterations and the adverse effects of cancer therapy, the majority of patients with GC experience malnutrition [2]. Sarcopenia, a progressive and generalized skeletal muscle disorder, is an independent predictor of adverse clinical outcomes in patients with GC [3, 4]. Therefore, the early and accurate identification of sarcopenia is essential for optimizing patient risk stratification and facilitating timely, individualized nutritional interventions.

The conventional diagnosis of sarcopenia involves assessing muscle strength, muscle quantity or quality and physical performance. This process is labour‐intensive and time‐consuming, limiting its application in routine clinical practice [5]. Currently, quantification of the skeletal muscle index (SMI) at the third lumbar vertebral (L3) level is widely accepted as a reference standard for assessing muscle mass, with robust evidence demonstrating its associations with clinical outcomes and overall survival across diverse malignancies [6, 7, 8]. Furthermore, recent studies have demonstrated high consistency between L3 level SMI and multislice or volumetric assessments, underscoring that muscle tissue at the L3 level harbours rich morphological and textural information extending far beyond mere cross‐sectional area [9, 10].

Radiomics has emerged as a promising approach for extracting high‐dimensional quantitative features from complex muscle textures [11]. Several studies have applied radiomics signatures from computed tomography (CT) images at different anatomical levels to predict sarcopenia and related clinical outcomes [12, 13, 14]. Alongside the development of radiomics, the integration of deep learning has advanced body composition analysis. Compared with traditional manual approaches, deep learning algorithms enable the automated and accurate segmentation of skeletal muscle, reducing interobserver variability and improving computational efficiency [15, 16, 17]. Furthermore, advanced deep learning models have shown robust performance in characterizing muscle phenotypes and directly predicting survival outcomes, linking radiological data with prognostic information [18, 19].

Currently, some studies define sarcopenia solely on the basis of low SMI, without incorporating other established measures such as muscle strength or physical performance, which may not fully capture the multidimensional nature of the condition. Moreover, the potential of a multimodal fusion model integrating clinical variables, radiomics features and deep learning features remains largely unexplored. This study aimed to develop and validate the sarcopenia model by combining radiomics and deep learning features with clinical variables to improve identification accuracy while reducing the manual workload in clinical practice.

## 2 Methods

### 2.1 Patient data

**FIGURE 1:**
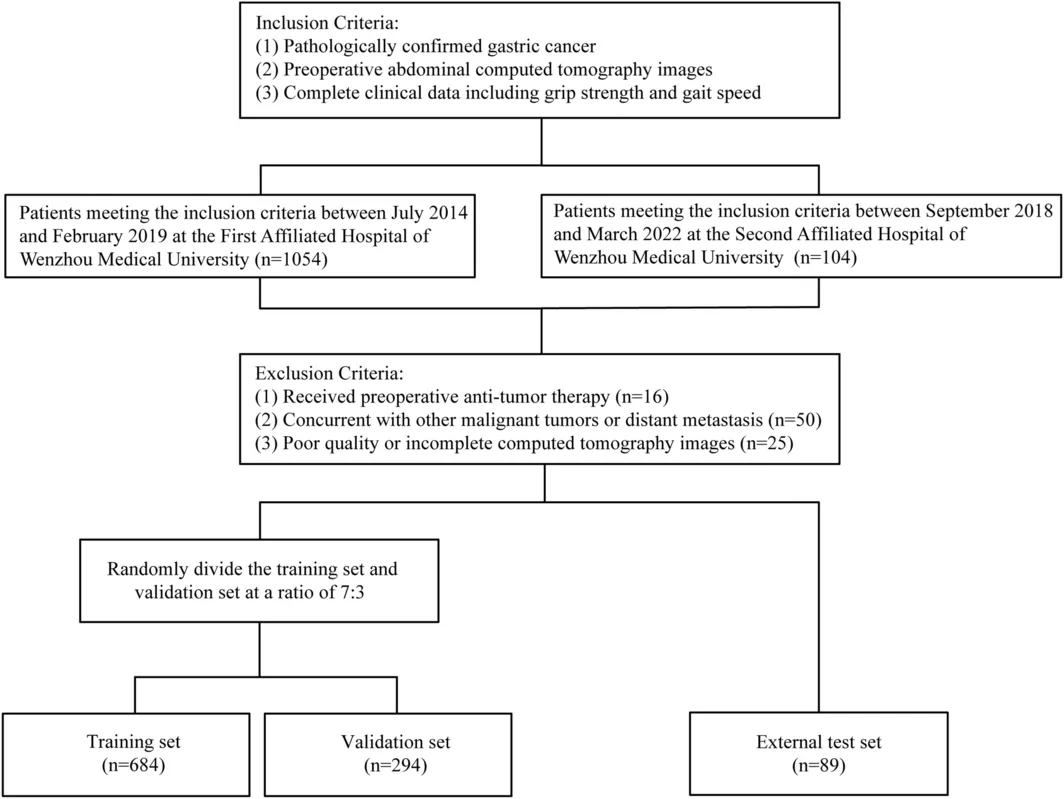
Flowchart of patient selection.

This study was a retrospective analysis of prospectively collected cohort data using radiomics and deep learning approaches and was approved by the institutional review board of the First Affiliated Hospital of Wenzhou Medical University (No. KY2023‐R006). Between July 2014 and February 2019, 1054 patients who underwent GC surgery at the First Affiliated Hospital of Wenzhou Medical University (Centre 1) were initially considered. An external cohort of 104 patients who underwent GC surgery at the Second Affiliated Hospital of Wenzhou Medical University (Centre 2) between September 2018 and March 2022 served as an external test set. The inclusion criteria were: (1) pathologically confirmed GC; (2) preoperative abdominal CT images; and (3) complete clinical data including grip strength and gait speed. The exclusion criteria were as follows: (1) received preoperative antitumour therapy (*n* = 16); (2) concurrent with other malignant tumours or distant metastasis (*n* = 50); and (3) poor quality or incomplete CT images (*n* = 25). A total of 1067 patients were ultimately included, comprising 978 patients from Centre 1 and 89 patients from Centre 2. Patients from Centre 1 were randomly divided into training and validation sets at a ratio of 7:3, yielding 684 and 294 patients, respectively. Patients from Centre 2 comprised the external test set (Figure 1). Patients from Centre 1 were followed for at least 5 years to assess overall survival (OS). OS was defined as the time from surgery to death from any cause.

### 2.2 Definition of Sarcopenia

According to the European Working Group on Sarcopenia in Older People (EWGSOP2) criteria [5], sarcopenia was defined by the presence of both low muscle strength and low muscle quantity or quality. Severe sarcopenia was defined as the coexistence of low muscle strength, low muscle quantity or quality and low physical performance. The abdominal CT images included in this study were acquired using the scanning parameters summarized in Table S1 and were subsequently processed using TotalSegmentator for automated skeletal muscle segmentation [20]. Axial slices at the L3 level, as well as one adjacent slice above and below this level, were extracted. Skeletal muscle area (SMA) was measured on the L3 level image. SMI was derived by normalizing the SMA to the square of height (cm^2^/m^2^) and was used as a measure of muscle mass. Low SMI was defined as < 40.8 cm^2^/m^2^ for men and < 34.9 cm^2^/m^2^ for women [21]. Muscle strength was assessed using grip strength, with cutoff values defined as < 28 kg for men and < 18 kg for women. Physical performance was assessed using the 6‐m gait speed test. A gait speed of < 1.0 m/s was defined as low physical performance [22].

### 2.3 Development of the Radiomics Model

Radiomics features were extracted from L3 level CT images using the PyRadiomics library. Images were resampled to a voxel spacing of 1 × 1 × 1 mm^3^ using nearest neighbour interpolation. Feature classes included shape, first‐order intensity and texture features, resulting in a total of 1288 features. The extracted radiomics features were standardized using *Z*‐score normalization. Spearman correlation analysis was then performed to identify features with high correlation, and those with correlation coefficients greater than 0.9 were removed. Feature selection was conducted using the least absolute shrinkage and selection operator (LASSO) regression with 10‐fold cross‐validation. An extreme gradient boosting (XGBoost) model was trained using the training set, with its hyperparameters optimized through grid search and class weights adjusted to account for class imbalance. The model's performance in classifying sarcopenia was assessed on the training, validation and external test sets. To interpret feature contributions and identify the most important predictors, SHapley Additive exPlanations (SHAP) analysis was performed on the trained model.

### 2.4 Development of 2D and 2.5D Deep Learning Models and the XGBoost Model

For the 2D deep learning model, the skeletal muscle slice at the L3 level was used as the input. For the 2.5D deep learning model, three consecutive muscle slices, including the L3 level and the adjacent upper and lower slices, were combined as a three channel input. Both the 2D and 2.5D deep learning models were constructed based on the ResNet50 architecture. All input images were resized to 224 × 224 pixels. Data augmentation strategies, including random horizontal and vertical flipping, were implemented to improve model generalizability. A ResNet50 architecture pretrained on the ImageNet dataset was used. Transfer learning was performed on the training set. During training, network parameters were iteratively updated using backpropagation with the Adam optimizer. The initial learning rate was set to 1 × 10^−3^, and the batch size was 64. The maximum number of training epochs was 100. Early stopping was applied to prevent overfitting. The trained models yielded the probability of sarcopenia based on the input images. After training, 2048 deep learning features were extracted from the penultimate global average pooling layer of the ResNet50 network for each input sample. In addition, an XGBoost‐based model was developed by integrating clinical variables, including gender, age, body mass index (BMI), haemoglobin and albumin, with radiomics features and 2.5D deep learning features. To ensure methodological consistency, the combined feature set underwent the same feature selection and model construction procedures as those used for the radiomics model.

### 2.5 Development of the Sarcopenia Model

**FIGURE 2:**
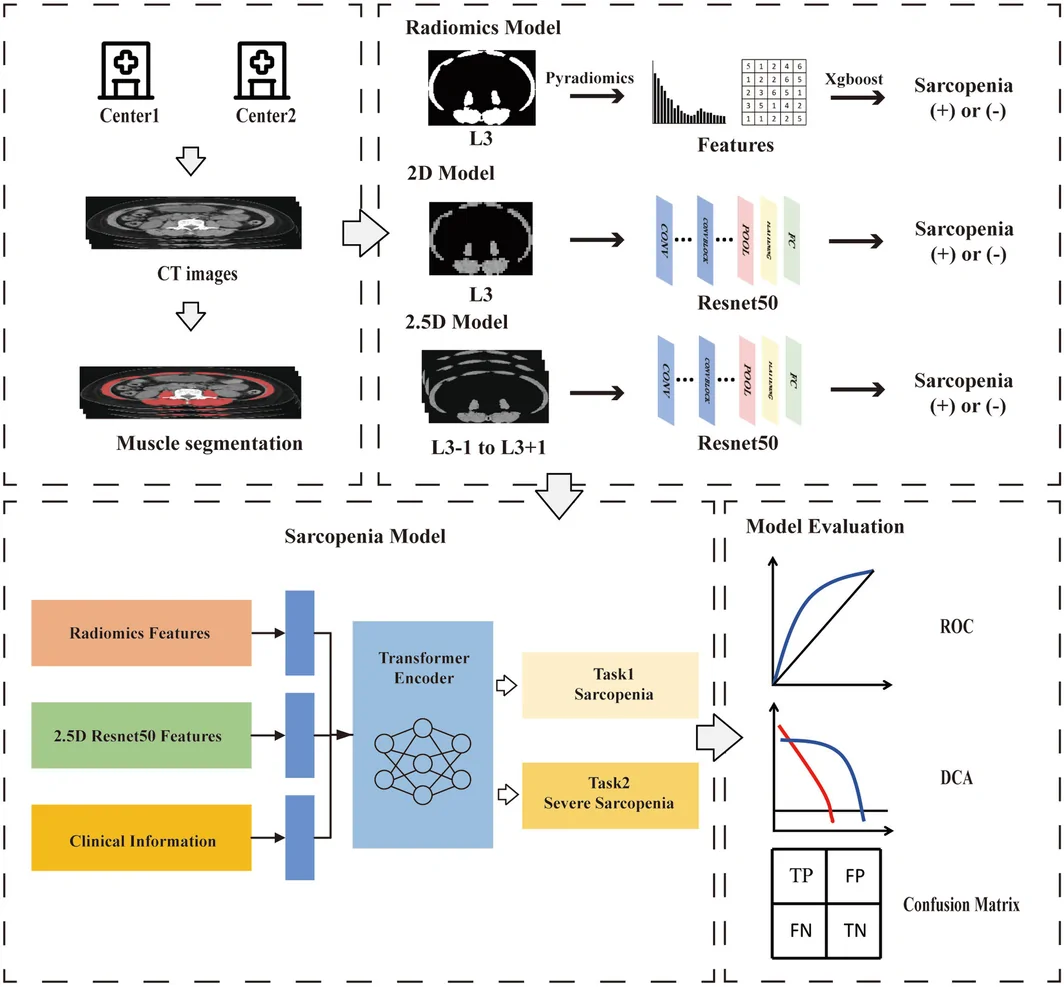
Overview of the study workflow.

To integrate clinical variables (including gender, age, BMI, haemoglobin and albumin), radiomics features and 2.5D deep learning features, a transformer‐based multimodal model was developed. This model allows complementary information across heterogeneous data modalities to be jointly exploited. Clinical, radiomics and deep learning features were considered as three independent input modalities. After normalization, features from each modality were separately fed into modality‐specific encoding networks composed of multiple fully connected hidden layers to learn high‐dimensional discriminative representations. Dropout regularization was applied during the encoding process to reduce overfitting. The encoded features from the three modalities were first projected into a 64‐dimensional latent space and then fused using a transformer encoder with two encoder layers and eight attention heads. The model's output consisted of two independent classification heads for estimating the probabilities of sarcopenia and severe sarcopenia, respectively (Figure 2). Detailed parameters are provided in Table S2.

### 2.6 Statistical Analysis

Model development and implementation were performed using Python (Version 3.7.13). All statistical analyses were conducted using R (Version 4.4.3) and the scikit‐learn library in Python. Baseline characteristics were summarized and compared using the compareGroups package in R. Categorical variables were compared using the chi‐square test or Fisher's exact test, while continuous variables were analysed using the independent Student's *t*‐test or the Mann–Whitney *U* test, as appropriate. Survival curves for patients with and without sarcopenia were estimated using the Kaplan–Meier method and compared using the log‐rank test. The predictive performance of the models was evaluated using receiver operating characteristic (ROC) curve analysis, and the area under the curve (AUC), accuracy, sensitivity and specificity were calculated. The 95% confidence intervals for the AUCs were estimated using 1000 bootstrap resamples. Additionally, decision curve analysis (DCA) was performed to assess the clinical net benefit of each model across a range of threshold probabilities. All statistical tests were two sided, and a *p* value < 0.05 was considered statistically significant.

## 3 Results

### 3.1 Patient Characteristics and Survival Outcomes

**FIGURE 3:**
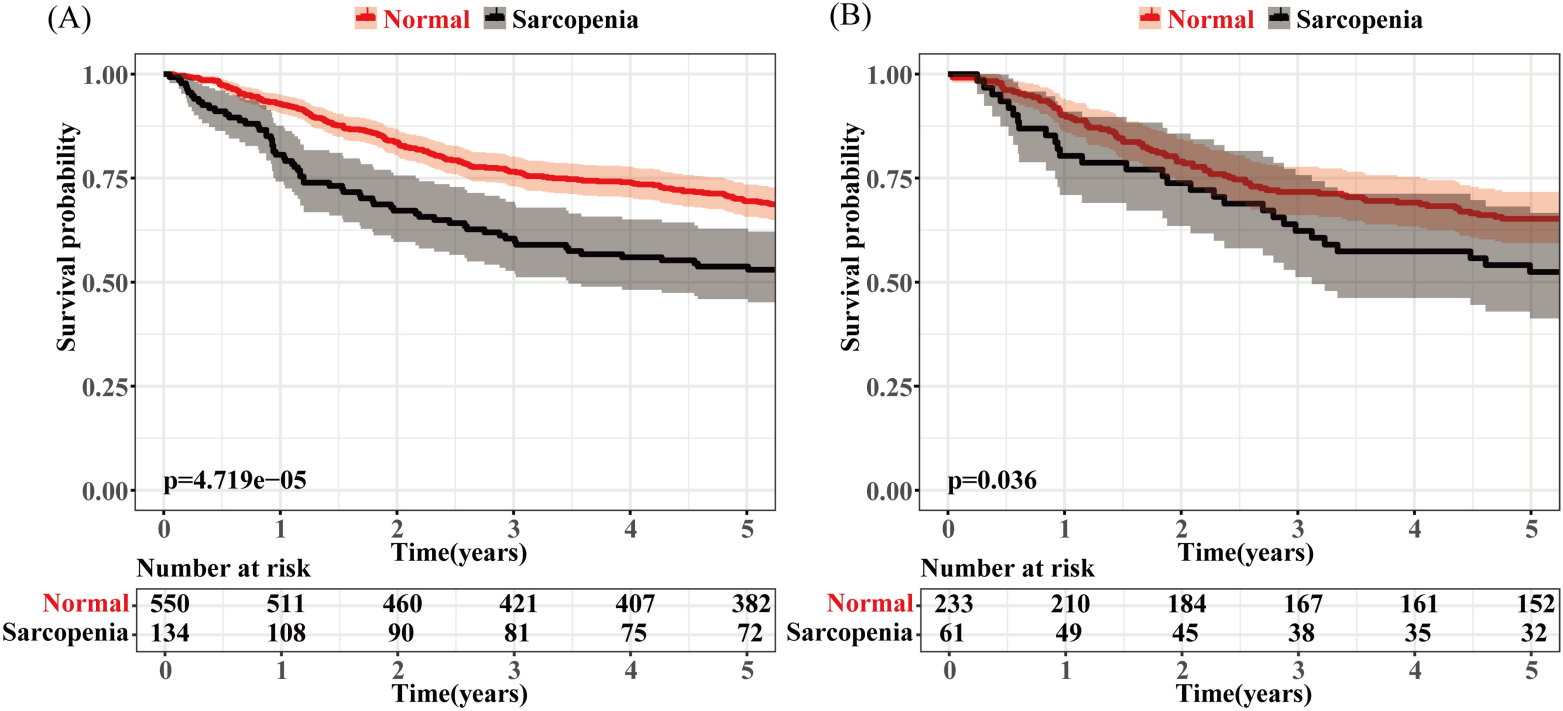
Kaplan–Meier survival curves comparing overall survival between normal and sarcopenic patients across different sets. (A) Overall survival of patients with sarcopenia versus normal patients in the training set. (B) Overall survival of patients with sarcopenia versus normal patients in the validation set.

A total of 1067 patients with GC were included in this study, comprising a training set (*n* = 684), a validation set (*n* = 294) and an external test set (*n* = 89). The baseline clinicopathological characteristics of the participants across the three sets are summarized in Table 1. The majority of the study population was male (73.3%), with a mean age of 64.3 ± 10.7 years and a mean BMI of 22.7 ± 3.0 kg/m^2^. No significant differences were observed among the training, validation and external test sets regarding gender, BMI, haemoglobin, albumin, grip strength, gait speed or SMI levels (*p* > 0.05). The prevalence of sarcopenia and severe sarcopenia was 20.0% (*n* = 213) and 12.3% (*n* = 131), respectively, and these proportions remained consistent across all sets (*p* > 0.05). Among patients from Centre 1, Kaplan–Meier survival analysis was performed to assess the prognostic impact of sarcopenia. In both the training and validation sets, patients with sarcopenia had significantly poorer OS than those without sarcopenia (log‐rank test, *p* < 0.05; Figure 3).

**TABLE 1:** Patient characteristics.

	All *N* = 1067	Training set *n* = 684	Validation set *n* = 294	Test set *n* = 89	*p*
**Gender**					0.34
Female	285 (26.7%)	174 (25.4%)	88 (29.9%)	23 (25.8%)	
Male	782 (73.3%)	510 (74.6%)	206 (70.1%)	66 (74.2%)	
**Age (year)**	64.3 (10.7)	65.0 (10.8)	63.0 (10.5)	64.0 (10.7)	0.03
**BMI (kg/m** ^**2**^ **)**	22.7 (3.0)	22.6 (3.0)	22.6 (3.1)	22.9 (3.0)	0.65
**Haemoglobin (g/L)**	120.8 (24.0)	121.0 (24.1)	121.4 (23.1)	118.0 (26.5)	0.55
**Albumin (g/L)**	38.3 (5.3)	38.1 (5.0)	38.6 (6.2)	38.7 (4.5)	0.23
**Grip strength (kg)**	28.0 (8.9)	27.9 (8.8)	27.9 (9.2)	29.4 (9.1)	0.32
**Gait speed (m/s)**	1.0 (0.3)	1.1 (0.3)	1.0 (0.2)	1.1 (0.3)	0.37
**SMI (cm** ^**2**^ **/m** ^**2**^ **)**	41.4 (7.8)	41.4 (7.6)	41.4 (8.1)	41.1 (8.0)	0.94
**TNM stage**					0.57
I	400 (37.5%)	249 (36.4%)	111 (37.8%)	40 (44.9%)	
II	229 (21.5%)	149 (21.8%)	61 (20.7%)	19 (21.3%)	
III	438 (41.0%)	286 (41.8%)	122 (41.5%)	30 (33.7%)	
**Sarcopenia**					0.92
No	854 (80.0%)	550 (80.4%)	233 (79.3%)	71 (79.8%)	
Yes	213 (20.0%)	134 (19.6%)	61 (20.7%)	18 (20.2%)	
**Severe sarcopenia**					0.58
No	936 (87.7%)	605 (88.5%)	253 (86.1%)	78 (87.6%)	
Yes	131 (12.3%)	79 (11.5%)	41 (13.9%)	11 (12.4%)	

*Note:* Data are presented as mean (SD) for continuous variables and as *n* (%) for categorical variables.

Abbreviations: BMI, body mass index; SMI, skeletal muscle index; TNM stage, tumour node metastasis stage.

### 3.2 Performance Comparison of the Radiomics, 2D, 2.5D and XGBoost Models

**FIGURE 4:**
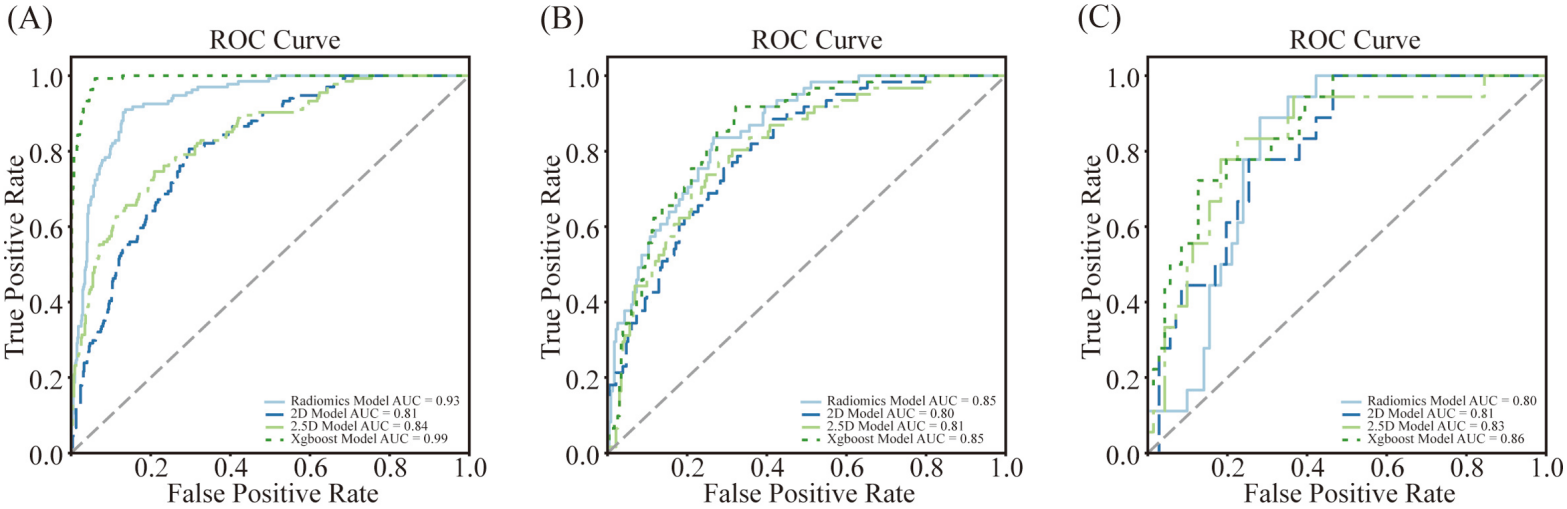
Receiver operating characteristic (ROC) curves comparing the diagnostic performance of the radiomics model, 2D model, 2.5D model and XGBoost model for sarcopenia. (A) ROC curves in the training set. (B) ROC curves in the validation set. (C) ROC curves in the external test set.

Feature selection yielded 26 sarcopenia‐related radiomics features and 56 multimodal features for the radiomics and XGBoost models, respectively. SHAP analysis was performed to quantify feature contributions in both models (Figures S1 and S2). In the XGBoost model, the leading SHAP features spanned all three modalities, and several were significantly correlated with grip strength and SMI, supporting their clinical and biological relevance (Figure S3). In parallel, 2D and 2.5D deep learning models were developed using the ResNet50 architecture. The radiomics, 2D, 2.5D and XGBoost models were compared using ROC analysis (Figure 4).

In the training set, the AUCs of the radiomics, 2D, 2.5D and XGBoost models were 0.93, 0.81, 0.84 and 0.99, respectively. In the validation set, the corresponding AUCs were 0.85, 0.80, 0.81 and 0.85, while in the external test set they were 0.80, 0.81, 0.83 and 0.86, respectively. Compared with the 2D model, the 2.5D model achieved a higher AUC, accuracy and specificity in the external test set, although its sensitivity was slightly lower. Given the more balanced performance, the 2.5D deep learning features were selected for subsequent multimodal fusion. Detailed performance metrics for all models across the three sets were summarized in Table 2.

**TABLE 2:** Performance of the radiomics, 2D, 2.5D and XGBoost models for predicting sarcopenia in gastric cancer patients.

Model name	AUC	95% CI	Accuracy	Sensitivity	Specificity
**Training set**					
Radiomics model	0.93	0.914–0.953	0.87	0.91	0.86
2D model	0.81	0.775–0.847	0.72	0.81	0.70
2.5D model	0.84	0.802–0.877	0.78	0.75	0.79
XGBoost model	0.99	0.986–0.996	0.95	0.99	0.94
**Validation set**					
Radiomics model	0.85	0.805–0.895	0.79	0.66	0.82
2D model	0.80	0.749–0.855	0.69	0.79	0.66
2.5D model	0.81	0.744–0.866	0.77	0.69	0.79
XGBoost model	0.85	0.803–0.898	0.82	0.62	0.87
**External test set**					
Radiomics model	0.80	0.700–0.886	0.74	0.50	0.80
2D model	0.81	0.709–0.903	0.66	0.78	0.63
2.5D model	0.83	0.713–0.921	0.80	0.72	0.82
XGBoost model	0.86	0.768–0.941	0.81	0.56	0.87

Abbreviations: AUC, area under the curve; CI, confidence interval.

### 3.3 Performance of the Sarcopenia Model

**FIGURE 5:**
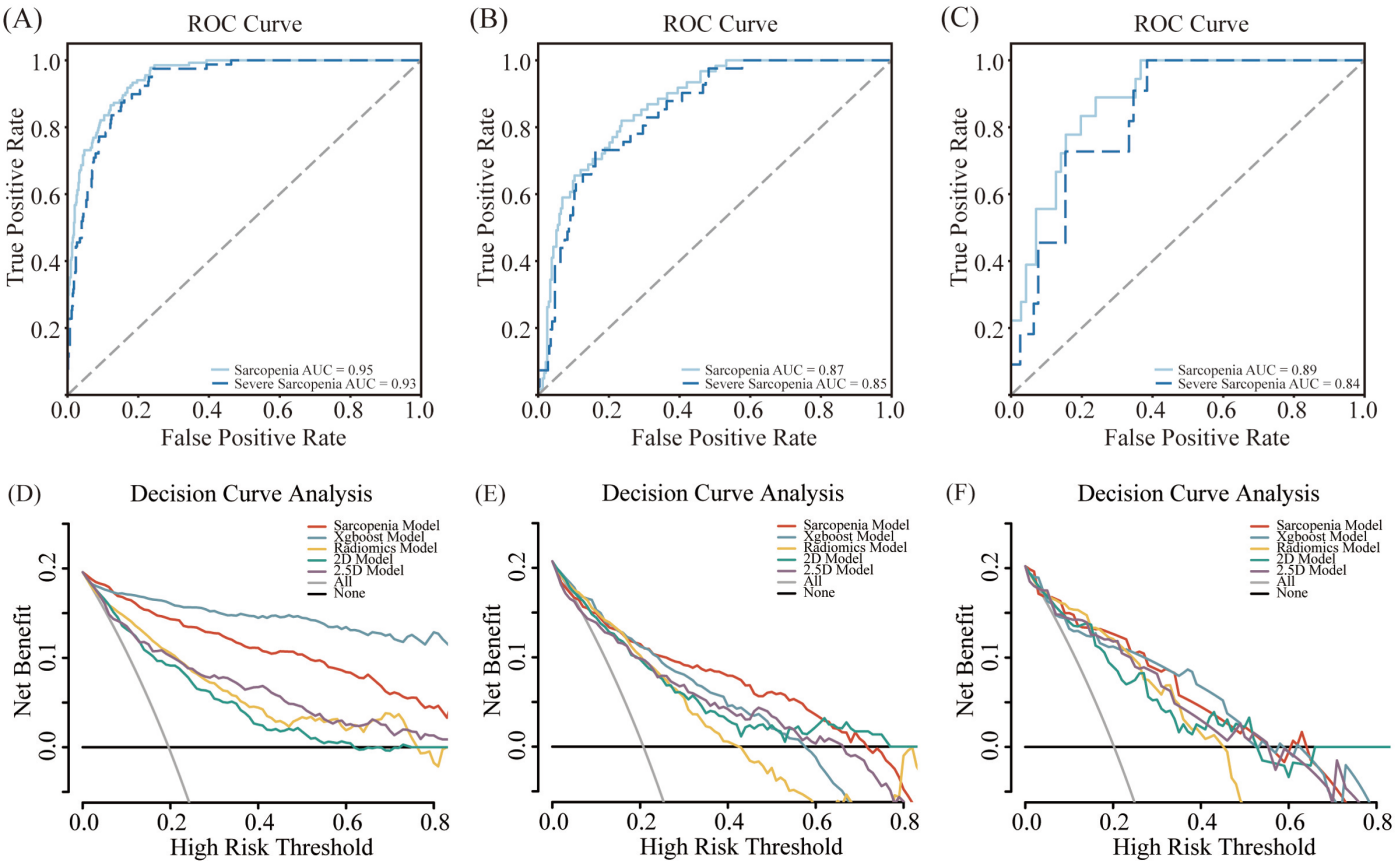
Receiver operating characteristic (ROC) curves and decision curve analysis (DCA) for evaluating the performance and clinical utility of the sarcopenia model. ROC curves for sarcopenia and severe sarcopenia in the training set (A), validation set (B) and external test set (C). DCA comparing the net benefit of the sarcopenia model with the radiomics model, 2D model, 2.5D model and XGBoost model in the training set (D), validation set (E) and external test set (F).

To integrate clinical variables (including gender, age, BMI, haemoglobin and albumin), radiomics features and 2.5D deep learning features, a sarcopenia model was developed. For sarcopenia, the AUCs were 0.95 (95% CI: 0.934–0.965), 0.87 (95% CI: 0.823–0.914) and 0.89 (95% CI: 0.807–0.952) in the training, validation and external test sets, respectively. For severe sarcopenia, the AUCs were 0.93 (95% CI: 0.904–0.953), 0.85 (95% CI: 0.792–0.901) and 0.84 (95% CI: 0.732–0.932). The performance metrics of the sarcopenia model across all sets are summarized in Table 3 and Figure 5. The DCA showed that the sarcopenia model provided a positive net benefit across clinically relevant threshold probabilities (Figure 5). Confusion matrices for sarcopenia and severe sarcopenia in the training, validation and external test sets are shown in Figure S4.

**TABLE 3:** Performance of the model in predicting sarcopenia and severe sarcopenia in gastric cancer patients.

Model name	AUC	95% CI	Accuracy	Sensitivity	Specificity
**Training set**					
Sarcopenia	0.95	0.934–0.965	0.84	0.93	0.82
Severe sarcopenia	0.93	0.904–0.953	0.78	0.97	0.76
**Validation set**					
Sarcopenia	0.87	0.823–0.914	0.77	0.80	0.76
Severe sarcopenia	0.85	0.792–0.901	0.72	0.78	0.71
**Test set**					
Sarcopenia	0.89	0.807–0.952	0.80	0.83	0.79
Severe sarcopenia	0.84	0.732–0.932	0.73	0.73	0.73

Abbreviations: AUC, area under the curve; CI, confidence interval.

## 4 Discussion

In this study, we developed and validated the sarcopenia model based on multimodal data fusion, incorporating clinical variables, radiomics features and 2.5D deep learning features to enable the automated assessment and risk stratification of sarcopenia in patients with GC. In the external test set, the model achieved an AUC of 0.89 for sarcopenia and 0.84 for severe sarcopenia, indicating good discriminative performance. Although the prognostic significance of sarcopenia in GC has been well established [21], current clinical assessment remains largely dependent on labour‐intensive procedures, including manual CT‐based muscle quantification by radiologists and clinician‐administered functional measurements such as grip strength and gait speed, which limits efficiency and scalability in routine practice. By establishing an automated workflow, this study facilitates the integration of high‐dimensional radiological data with clinical information and may provide a feasible approach for clinical screening and risk stratification.

Tumour‐associated chronic inflammation disrupts protein metabolism and ultimately impairs patients' ability to tolerate and adapt to surgical stress. In this context, muscle wasting represents more than a simple reduction in body weight and reflects an underlying global physiological vulnerability [23, 24]. As these pathophysiological alterations directly influence postoperative recovery and long‐term prognosis, comprehensive risk stratification has become central to personalized prehabilitation [25]. In our cohort, Kaplan–Meier survival analysis showed a significant difference in long‐term survival between patients with and without sarcopenia (*p* < 0.05), consistent with findings from recent studies in GC [26]. Accordingly, the proposed model may serve as an early screening tool for sarcopenia in routine clinical practice, helping to identify high‐risk patients who may benefit from further functional assessment and earlier targeted prehabilitation and nutritional interventions.

The sarcopenia model integrates clinical variables, radiomics features and 2.5D deep learning features, with each modality contributing a distinct yet complementary role in characterizing this complex phenotype. Clinical variables such as age, BMI and albumin provide essential macro‐level context by reflecting the patient's baseline physiological condition and systemic nutritional status [27]. Simultaneously, radiomics features are instrumental in quantifying textural heterogeneity and muscle quality that are often imperceptible to the human eye [28]. Previous studies have demonstrated that radiomics features extracted at different anatomical levels can be leveraged to predict sarcopenia or survival outcomes across multiple cancer types [29, 30]. Beyond radiomics‐based approaches, other studies have highlighted the potential of deep learning by utilizing CT images or chest radiographs to predict clinical outcomes, including metastasis, survival and sarcopenia [18, 31]. The sarcopenia model incorporates specialized deep learning features as a core component of the multimodal fusion. We specifically utilized 2.5D model features with superior AUC values as input data because they effectively capture the critical anatomical spatial continuity across adjacent slices while avoiding the inherent limitations of isolated 2D analysis. By integrating these distinct data streams, the sarcopenia model may reduce the influence of noise and variability within individual feature sets. This holistic approach helps ensure that the model maintains robust generalizability and reliable discrimination across both validation and external test sets. The potential value of multimodal integrated frameworks has also been reported in other challenging oncological tasks, including the prediction of occult lymph node metastasis in laryngeal squamous cell carcinoma and the identification of microsatellite instability in colorectal cancer [32, 33].

A major strength of this study is that the reference labels were defined according to clinical diagnostic criteria incorporating muscle mass, muscle strength and physical performance, rather than CT‐derived muscle area alone. Compared with studies that define sarcopenia solely by SMI, this approach better reflects the combined structural and functional nature of sarcopenia [30, 34]. We implemented a dual‐output system to simultaneously identify sarcopenia and severe sarcopenia. Although the diagnostic performance for severe sarcopenia was slightly lower than that for sarcopenia, this difference likely reflects the inherent biological complexity at the interface between structural muscle depletion and functional decline. As an integral component of the diagnostic criteria for severe sarcopenia, gait speed represents a highly integrative functional measure that depends not only on muscle structure but also on multiple other factors, including comorbid conditions, neuromuscular coordination and cardiorespiratory function. Consequently, functional performance in patients with severe sarcopenia exhibits greater heterogeneity, posing a more challenging scenario for algorithmic modelling based on imaging‐derived features. Further misclassification analysis showed that incorrectly classified cases differed from correctly classified cases in several clinical and sarcopenia‐related characteristics (Tables S3 and S4), suggesting that differences in nutritional status, muscle quantity and physical function may influence model discrimination. In this context, the model outputs may still provide clinically informative signals that complement routine assessment. In practice, these results can help clinicians identify patients with greater functional vulnerability, prompt closer functional evaluation and support more individualized perioperative care planning.

Despite these promising results, several limitations of this study warrant consideration. First, although this study represents a retrospective re‐analysis of prospectively collected data, further validation in large scale prospective cohorts is required to confirm the model's real‐world predictive performance and to support further optimization of severity stratification, including the reduction of false positive predictions for severe sarcopenia. Second, our analysis focused on specific muscle regions across three representative anatomical slices. Incorporating volumetric data from a broader range of abdominal levels may provide a more comprehensive assessment of systemic muscle wasting. Finally, while we strictly followed the standardized clinical consensus for sarcopenia diagnosis, our study relied primarily on CT‐based assessments and did not incorporate measurements from other established modalities such as bioelectrical impedance analysis or dual‐energy x‐ray absorptiometry. As a result, the generalizability of the model to clinical settings that rely on different body composition assessment technologies may be limited.

## 5 Conclusions

In summary, we developed the sarcopenia model integrating clinical variables, radiomics features and 2.5D deep learning features. By simplifying and optimizing the evaluation workflow, the model enables the automated identification of patients with GC at high risk of sarcopenia, thereby reducing reliance on labour‐intensive manual assessments and providing a basis for earlier implementation of individualized nutritional and functional interventions in routine clinical practice.

## Conflicts of Interest

The authors declare no conflicts of interest.

## Supporting information


**Table S1:** CT acquisition parameters.
**Table S2:** Parameters of the sarcopenia model.
**Table S3:** Characteristics of misdiagnosed cases in sarcopenia prediction.
**Table S4:** Characteristics of misdiagnosed cases in severe sarcopenia prediction.
**Figure S1:** Feature selection and interpretability analysis for the radiomics model.
**Figure S2:** Feature selection and interpretability analysis for the XGBoost model.
**Figure S3:** Correlations between XGBoost model features and sarcopenia‐related clinical indicators in the training, validation and external test sets.
**Figure S4:** Confusion matrices of the sarcopenia model for predicting sarcopenia and severe sarcopenia in the training, validation and external test sets.

## Data Availability

The data that support the findings of this study are available from the corresponding author upon reasonable request.
