# Supplementary material for: Integrated Assessment of Sarcopenia in Patients with Gastric Cancer Using Deep Learning and Radiomics
Source: J Cachexia Sarcopenia Muscle. 2026 Sep 14;17(5):e70377. doi: 10.1002/jcsm.70377 (PMC13574536; doi:10.1002/jcsm.70377)
Supplement: Supplementary file 1 [file JCSM-17-e70377-s001.docx]

**Integrated Assessment of Sarcopenia in Patients with Gastric Cancer Using Deep Learning and Radiomics**

Huaiqing Zhi^1^, Jingwei Zheng^1^, Hao Chen^1^, Xinxin Yang^5^, Zhixuan Jiang^1^, Ang Shi^1^, Dongze Wu^1^, Linxi Lv^1^, Jinnuo He^1^, Xiaodong Chen^1^, Bujian Pan^2^, Xian Shen^1,3,4^, Weiteng Zhang^1^

Supplementary Table 1 CT acquisition parameters

|  | Center 1 | Center 2 |
| --- | --- | --- |
| **CT Type** | Non-contrast CT | Non-contrast CT |
| **Tube Voltage** | 100-140 kVp | 120 kVp |
| **Tube Current** | 84-596 mA | 149-596 mA |
| **Rotation Time** | 0.5-1.0 s | 0.5-0.8 s |
| **Pixel spacing** | approximately 0.70 × 0.70 mm² | approximately 0.68 × 0.68 mm² |
| **Matrix** | 512 x 512 | 512 x 512 |

Supplementary Table 2 Parameters of the sarcopenia model

|  | **Input feature** | **Hidden units** | **Dropout rate** |
| --- | --- | --- | --- |
| **Clinical** | gender, age, BMI, hemoglobin, and albumin | 16, 32 | 0.1 |
| **Radiomics** | Radiomics features | 512, 256, 128 | 0.4 |
| **Deep learning** | 2.5D ResNet50 features | 1024, 512, 256, 128 | 0.5 |

Supplementary Table 3 Characteristics of misdiagnosed cases in sarcopenia prediction

|  | misdiagnosed | Correct | P value |
| --- | --- | --- | --- |
| **Number of patients** | 195 | 872 |  |
| **Age (year)** | 68.00 [62.50, 74.00] | 64.00 [57.00, 72.00] | <0.001 |
| **BMI (kg/m^2^)** | 21.99 [19.91, 23.69] | 22.86 [20.70, 24.57] | <0.001 |
| **Hemoglobin (g/L)** | 117.00 [99.00, 130.00] | 127.00 [108.00, 140.00] | <0.001 |
| **Albumin (g/L)** | 38.00 [34.80, 40.85] | 38.90 [35.30, 41.70] | 0.028 |
| **SMI (cm^2^/m^2^)** | 36.62 [32.56, 40.35] | 42.54 [36.88, 47.80] | <0.001 |
| **Grip Strength (kg)** | 24.70 [20.05, 30.75] | 28.65 [22.00, 35.50] | <0.001 |
| **Gait Speed (m/s)** | 1.00 [0.85, 1.15] | 1.04 [0.88, 1.21] | 0.022 |

Supplementary Table 4 Characteristics of misdiagnosed cases in severe sarcopenia prediction

|  | misdiagnosed | Correct | P value |
| --- | --- | --- | --- |
| **Number of patients** | 256 | 811 |  |
| **Age (year)** | 69.00 [63.00, 75.00] | 64.00 [56.00, 71.00] | <0.001 |
| **BMI (kg/m^2^)** | 21.62 [19.79, 23.63] | 22.95 [20.86, 24.62] | <0.001 |
| **Hemoglobin (g/L)** | 117.00 [98.00, 129.00] | 128.00 [110.00, 141.00] | <0.001 |
| **Albumin (g/L)** | 37.55 [34.30, 40.55] | 39.10 [35.70, 41.80] | <0.001 |
| **SMI (cm^2^/m^2^)** | 35.97 [32.54, 39.79] | 43.23 [37.88, 48.30] | <0.001 |
| **Grip Strength (kg)** | 22.85 [18.88, 28.83] | 29.90 [23.05, 36.00] | <0.001 |
| **Gait Speed (m/s)** | 1.04 [0.92, 1.20] | 1.02 [0.88, 1.20] | 0.349 |

**Supplementary Figure 1 Feature selection and interpretability analysis for the radiomics model.**

 (A) Least absolute shrinkage and selection operator (LASSO) coefficient profiles of radiomics features across different values of the regularization parameter (λ). (B) Ten-fold cross-validation curve showing the mean squared error for LASSO feature selection, with the optimal λ value indicated by the dashed vertical line. (C) Coefficients of the selected features retained in the radiomics model. (D) SHapley Additive exPlanations (SHAP) summary plot showing the top 20 features ranked by SHAP value, illustrating their relative contributions to the radiomics model output. Each point represents a feature value for an individual patient, with color indicating the feature magnitude.

**Supplementary Figure 2 Feature selection and interpretability analysis for the XGBoost model.**

(A) LASSO coefficient profiles of the combined multimodal features across different values of the regularization parameter (λ). (B) Ten-fold cross-validation curve showing the mean squared error for LASSO feature selection, with the optimal λ value indicated by the dashed vertical line. (C) Coefficients of the selected multimodal features retained in the XGBoost model. (D) SHapley Additive exPlanations (SHAP) summary plot showing the top 20 features ranked by SHAP value, illustrating their relative contributions to the XGBoost model output. Each point represents a feature value for an individual patient, with color indicating the feature magnitude.

**Supplementary Figure 3 Correlations between XGBoost model features and sarcopenia-related clinical indicators in the training, validation, and external test sets.**

The color scale represents the correlation coefficient, with red indicating positive correlations and blue indicating negative correlations. Asterisks indicate statistical significance: *P < 0.05, **P < 0.01, and ***P < 0.001.

**Supplementary Figure 4 Confusion matrices of the sarcopenia model for predicting sarcopenia and severe sarcopenia in the training, validation, and external test sets.**

Rows represent the true labels, and columns represent the predicted labels. Each cell shows the number of patients and its percentage within the corresponding cohort. Darker shading indicates a larger number of patients.
